# Predictive and Prognostic Role of Peripheral Blood T-Cell Subsets in Triple-Negative Breast Cancer

**DOI:** 10.3389/fonc.2022.842705

**Published:** 2022-02-15

**Authors:** Meng Li, Junnan Xu, Cui Jiang, Jingyan Zhang, Tao Sun

**Affiliations:** Department of Medical Oncology, Cancer Hospital of China Medical University, Liaoning Cancer Hospital & Institution, Shenyang, China

**Keywords:** triple-negative breast cancer, lymphocytes, CD4+ T cell, CD4+/CD8+ ratio, prognosis, biomarker, immune checkpoint inhibitor, chemotherapy

## Abstract

**Background:**

Triple-negative breast cancer (TNBC), as a highly aggressive and metastatic tumor, can still not contain the medical needs. It has become an urgent problem to develop prognostic markers further and realize precision medicine. The predictive and prognostic significance of peripheral blood lymphocytes, as well as the clinicopathological factors affecting them, were explored in the present study.

**Methods:**

The clinicopathological data of 278 patients with TNBC were collected and analyzed retrospectively. Peripheral blood lymphocytes (pBL) and blood routine indexes before treatment were quantified by flow cytometry analysis. Progression-free survival (PFS) and overall survival (OS) were analyzed by the Kaplan-Meier curve and Cox hazard proportion regression model. The associations between hematologic parameters and treatment response and clinicopathological characteristics were estimated by the Mann-Whitney test and Spearman test.

**Results:**

Compared with all blood routine indexes, only a significant correlation between better treatment efficacy and higher peripheral CD4 +/CD8 + ratio of TNBC patients was observed (P=0.059), particularly those treated with chemotherapy combined with immune checkpoint inhibitors (P=0.048). Among the pBL subsets, CD4 + T lymphocyte was the only independent factor that could predict the prognosis of metastatic TNBC. Patients presenting higher values of peripheral CD4 + T lymphocytes showed longer PFS (median PFS 9 months vs. 5 months; HR =0.65, 95%CI: 0.440-0.973, P = 0.032) and OS (median OS 31 months vs. 16 months; HR=0 .63, 95%CI: 0.417-0.940, P< 0.01). Especially CD4+ was found predictive for prognosis in TNBC patients who received chemotherapy (P<0.05). Finally, the older age, higher clinical stage, and more advanced treatment lines were related to the lower level of CD4 +. The older age and having received neoadjuvant therapy were related to the lower CD4 +/CD8 + ratio (P<0.05).

**Conclusion:**

The baseline CD4+/CD8+ cell ratio in peripheral blood is associated with therapeutic response, especially for chemotherapy combined with immunotherapy. Peripheral CD4+ cells can steadily predict all clinical outcomes for patients with mTNBC, and this clinical prognosis prediction is significantly related to chemotherapy. Peripheral CD4+ and CD4+/CD8+ are both closely associated with clinicopathological parameters.

## Introduction

Breast cancer is one of the most common malignancies in women. According to the latest world cancer data released by International Agency for Research on Cancer (IARC) in 2020, female breast cancer has surpassed lung cancer as the most common cancer (11.7%). Still, it is not the highest death rate for cancer (6.9%) (1). Triple-negative breast cancer (TNBC) is negative for estrogen receptor (ER), progesterone receptor (PR) and human epidermal growth factor receptor 2 (HER2), accounting for about 15%-20% of breast cancer. Early TNBC has the characteristics of high recurrence rate, high incidence of visceral metastasis, and short overall survival (2). Advanced TNBC is highly invasive, and the prognosis is often poor (3). Up to now, chemotherapy is still the cornerstone of TNBC treatment. However, the prognosis of patients is still far worse than that of other molecular subtypes of breast cancer, and new therapies are urgently needed. In 2018, based on the Olympia-D study and EMBRACA study, PARP inhibitors were approved by FDA for HER2 negative metastatic breast cancer with BRCA mutation (10%-20% mutation in TNBC patients) (4). Immune checkpoint inhibitors (ICIs) provided a breakthrough for cancer treatment. While breast cancer is usually not considered a highly immunogenic tumor, researchers found that TNBC is the most closely connected breast cancer subtype with the immune microenvironment. It has higher immunogenicity, higher enrichment by tumor-infiltrating lymphocytes (TILs), higher tumor mutation load (TMB), and higher levels of programmed cell death ligand 1 (PD-L1) expression. These suggest that patients with TNBC are more likely to respond to immunotherapy. The application of ICIs has achieved good results in the rescue treatment of advanced TNBC and neoadjuvant treatment of early TNBC (5). In addition, EGFR/PI3K/AKT/mTOR/CDK4/6 inhibitors, androgen receptor inhibitors, and ADC drugs are all explored. However, with the selection of targets, efficacy of targeted drugs, and side effects, the exploration of effective biomarkers has become a critical problem to be addressed (6).

The progression of breast cancer has a close correlation with the immune system. Innate immunity and adaptive immunity affect the occurrence, development, and metastasis of breast cancer. Immune tolerance is one of the fundamental reasons to explain tumor resistance to chemotherapy and immunotherapy. Cellular immunity plays a leading role in tumor immunity, especially lymphocyte subsets’ function is crucial for tumor immune monitoring (7). Available evidence suggests that the increase of TILs before treatment can improve the response to therapy and is related to the prolongation of survival, especially in the clinical TNBC subtype (7–10). At present, the International TILs Working Group has begun a standardized assessment of breast cancer TILs, identifying patients who may only need to use new immunotherapy (including checkpoint inhibitor therapy) and providing the best combination and timing for these effective treatments (11). Previous studies primarily focused on the local immune response in the tumor microenvironment. Still, many studies showed that the local anti-tumor immune response independent of the systemic immune system does not exist. Clinical studies have shown that the key factor for the improvement of patients in the process of cancer immunotherapy may be the presence of unexpended T cells and T cell clones in peripheral blood (12). Monitoring and regulating tumor treatment through the overall peripheral blood immune state has become a hot spot. However, very little was found in the literature on the predictive and prognostic value of PBL. In this case, we focused on peripheral blood lymphocytes, which have several advantages of simple operation, easy access, low invasiveness, dynamic monitoring, and high homogeneity compared with TILs. In addition, many clinical studies of breast cancer have shown that the neutrophil-to-lymphocyte ratio (NLR) and the platelet-to-lymphocyte ratio (PLR) before treatment are associated with the responsiveness and prognosis, and the disease-specific outcome in TNBC has a more significant impact (13–15). To study the overall immune environment of tumors, we analyzed lymphocyte parameters and blood routine indexes in circulating blood of TNBC to provide essential data and reference basis for exploring biomarkers with rapid response and easy identification to help guide treatment decisions.

## Data and Methods

### Clinical Data

A total of 278 patients with TNBC followed at Liaoning Cancer Hospital & Institute from 2010.7 to 2021.7 were enrolled in this retrospectively study. Cases were screened according to the following criteria, inclusion criteria: complete pathological data of patients confirmed that the expression of estrogen receptor (ER), progesterone receptor (PR), and human epidermal growth factor receptor 2 (HER2) were negative by immunohistochemistry; clinicopathological and survival data were complete; Complete blood routine and lymphocyte immunoassay data were available within three months before and after treatment; Exclusion criteria: Clinicopathological data were confirmed as non-TNBC patients; clinical or pathological data was incomplete or loss of follow-up; people with autoimmune disease or take prior treatment that affects the immune function of the body (such as immune function enhancing drugs). The baseline clinical information of the study population included: age, family history, menstrual status, tumor diameter, lymph node metastasis, clinical stage, Ki-67 expression status, visceral metastasis, BRCA status, multiple metastases, treatment, etc. All patients were followed up by telephone. Up to the final follow-up time to 2021.7, 177 cases died, and the median follow-up time was 24 months. The ethics committee of our institution approved the current study with the ethics number 20201133K.

### Study Endpoint and Prognosis Evaluation

The primary endpoints of this study were therapeutic response, progression-free survival (PFS), and overall survival (OS). Efficacy determination: after two cycles of treatment, treatment responses including complete response (CR), partial response (PR), stable disease (SD), and progressive disease PD were determined with Response Evaluation Criteria in Solid Tumors, RECIST version 1.1, and immunotherapy responses were determined according to i-RECIST v1.1 criteria. PFS was defined as the time interval between the date of patient registration and the date of first documented disease progression or the time interval of death before PD. OS was defined as the interval from patient registration to death or study deadline.

### Immunohistochemistry and Blood Sample Analysis

Multiple markers of breast tumors were detected by immunohistochemistry. Two pathologists performed readings independently and were not affected by clinical results. The average of the two evaluations was used for this analysis. All blood samples were obtained by peripheral venipuncture and placed in a test tube containing ethylenediaminetetraacetic acid (EDTA) and immediately sent for analysis. In our institute, all blood samples were taken after 8 hours of fasting. The counts of different lymphocyte subsets (CD3 +, CD8 +, CD4 +, CD19 +, CD56 +, CD5+, CD4 +/CD8 + ratio and Lymph Events) in peripheral blood were detected by antibody staining and flow cytometry. A series of monoclonal antibodies and flow cytometry were purchased from BD (BD Biosciences, Franklin Lake, NJ, USA) and operated according to the reagent instructions. The count of blood routine indexes (red blood cells count, hemoglobin, white blood cells count, lymphocytes, neutrophils, monocytes, and platelets) was counted by an automatic blood analyzer (xn-5000, Sysmex, Kobe, Japan). NLR and PLR were defined as the ratio of the neutrophil count and platelet count to lymphocyte count.

### Statistical Analysis

In the descriptive analysis, quantitative variables are described as mean and range, while qualitative variables are described as quantity and percentage. The Kaplan-Meier method was used to estimate PFS and OS. The Mantel-Cox method was used to generate hazard ratio (HR) and 95% confidence interval (CI) with difference detection by log-rank test. Spearman’s Rho was used to estimate the correlation, and the nonparametric Mann – Whitney U test was used to evaluate the comparison. The multivariate logistic regression model was used for multivariate analysis. All P values were two-tailed-sided, and statistical significance was set as P < 0.05. All analyses were performed using SPSS 26.0 software (Armonk, NY, USA) and GraphPad Prism 9.0 (GraphPad, Inc, San Diego, CA, USA).

## Results

### Baseline Characteristics and PBL Subset Distribution

**Figure 1:**
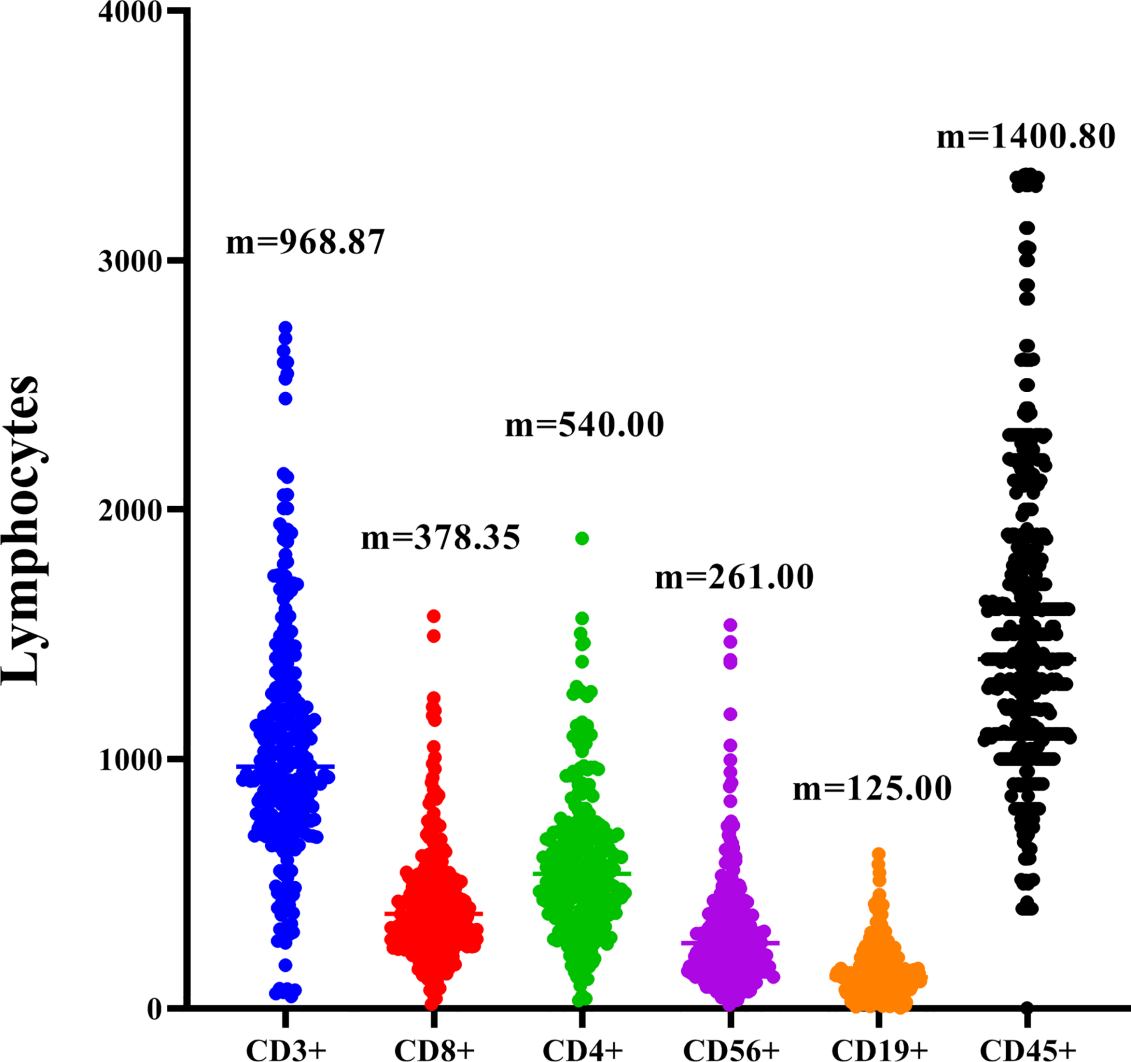
Distribution of peripheral blood CD3+, CD8+, CD4+, CD19+,CD45+ and CD56+ in TNBC. M is the median of each lymphocyte subpopulation.

**Figure 2:**
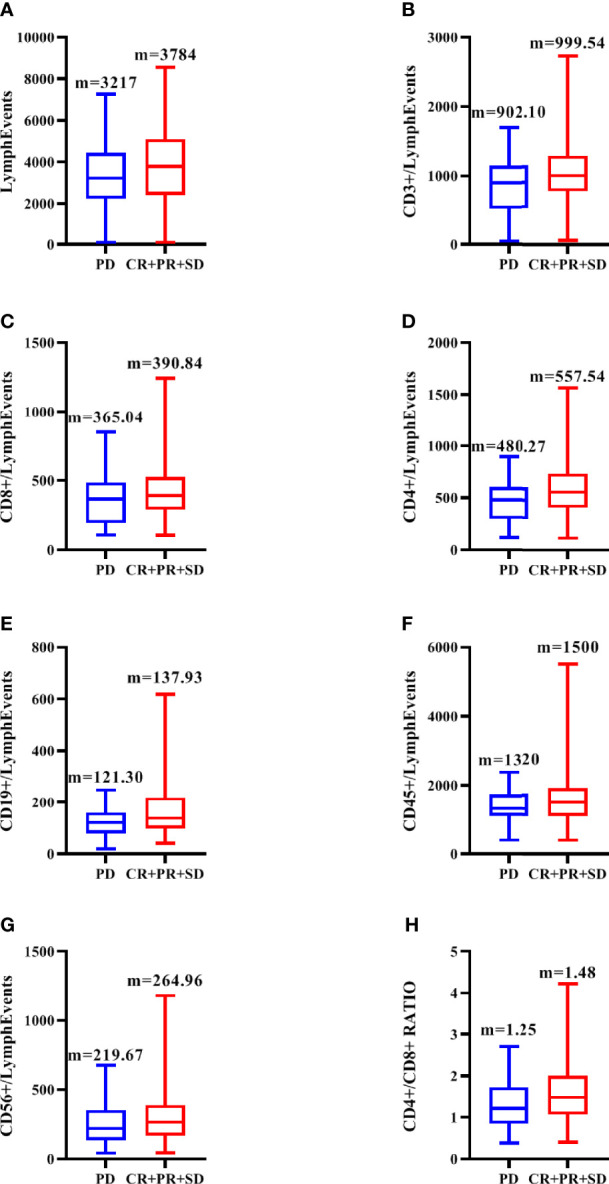
Distribution of peripheral blood total lymph events **(A)**, CD3+ **(B)**, CD8+ **(C)**, CD4+**(D)**, CD19+ **(E)**, CD45+ **(F)**, CD56+**(G)** and CD4+/CD8+**(H)** in CR/PR/SD subgroup and PD subgroup. M is the median of each lymphocyte subpopulation.

The 278 TNBC patients included in the study were women, and the median age was 65 years (22-83 years). **Table 1** showed the clinical and pathological data of all patients in detail. Blood routine indexes and peripheral blood lymphocyte subsets were evaluated at baseline. The distribution of lymphocyte subsets in peripheral blood was presented in **Figure 1**. All patients were classified according to disease control (CR, PR, and SD) and disease progression (PD). We observed that the levels of peripheral blood lymphocyte subsets of patients in the CR/PR/SD subgroup (n = 227) were higher than those in the PD subgroup (n = 51) (**Figure 2**).

**Table 1:** Main baseline clinicopathological characteristics of the study population (N=278).

Variables	N (%)
Age (y)	
≤50	145 (52.16%)
>50	133 (47.84%)
**Ki67**	
Positive (≥14%)	206 (74.10%)
Negative (<14%))	72 (25.90%)
**Pathologic T stage** (Tumor size)	
≤1	106 (38.13%)
2-4	172 (61.87%)
**Pathologic N stage**	
0	113 (40.65%)
1-2	118 (42.45%)
3	47 (16.91%)
**Treatment phase**	
Chemotherapy Neoadjuvant	7 (25.17%)
Postoperative adjuvant therapy	92 (33.09%)
First or second line Advanced treatment	148 (53.24%)
Multi-line Advanced treatment	31 (11.15%)
**Visceral invasion**	
Yes	91 (32.73%)
No	187 (67.26%)
**Multiple invasion**	
Yes	180 (64.75%)
No	98 (35.25%)
**Type of treatment**	
Chemotherapy only	244 (87.77%)
Chemotherapy combined with targeted therapy	20 (7.19%)
Chemotherapy combined with immunotherapy	14 (5.04%)
**Clinical stages**	
**0**	1 (0.36%)
1	67 (24.10%)
2	23 (8.27%)
3	26 (9.35%)
4	161 (57.92%)
**Adjuvant therapy**	
Yes	48 (17.27%)
No	230 (82.73%)
**Family tumor history**	
Yes	231 (83.09%)
No	47 (16.91%)
**BRCA status**	
mutation	4 (1.44%)
non-mutation	2 (0.72%)
unknown	272 (97.84%)
**Menstrual status**	
Premenopause	93 (33.45%)
Postmenopause	185 (66.55%)
**Curative effect**	
Therapy response	227 (81.65%)
Disease Progression	51 (18.35%)
**Living conditions**	
Survival	177 (63.67%)
Death	101 (36.33%)
**KPS**	
≥80	271 (97.48%)
50-70	7 (2.52%)

KPS, Karnofsky Performance Status.

### Correlation Between Hematologic Parameters and Treatment Response

Univariate analysis (**Table 2A**) showed that the values of CD3 + (P = 0.042), CD4 + (P = 0.004), and CD4 +/CD8 + ratio (P = 0.035) in CR/PR/SD group were significantly higher than those in PD group, P < 0.05 was included in multivariate analysis. In contrast, the relationship between blood routine indexes and clinical efficacy, which we considered to be of interest in this study, did not exhibit significant differences.

**Table 2:** Univariate (**A**) and multivariate analysis (**B**) for correlation of circulating lymphocytes with therapy response in TNBC.

A		
All Cases	Z	P
CD3+	-2.035	0.042
Lymphocytes	-1.45	0.147
CD8+	-0.015	0.988
CD4+	-2.919	0.004
CD56+	-1.8	0.072
CD19+	-0.57	0.568
CD45+	-1.954	0.051
CD4+/CD8+ Ratio	-2.106	0.035
WBC	-0.198	0.843
RBC	-0.244	0.807
PLT	-0.974	0.330
HB	-0.332	0.740
Neutrophils	-0.563	0.573
Lymphocytes	-0.977	0.329
NLR	-0.861	0.389
PLR	-1.113	0.266
**B**		
**All Cases**	**P**	**Odds ratio (95%CI)**
CD3+	0.295	1.679 (0.799-4.639)
CD4+	0.600	1.533 (0.311-1.965)
CD4+/CD8+ Ratio	0.059	1.873 (0.967-3.782)

95%CI, 95% confidence interval.

All P values were two-tailed-sided, and statistical significance was set as P < 0.1.

(Mann-Whitney U test and Multivariate logistic regression model).

In multivariate analysis (**Table 2B**), adjust the P value to 0.10, only CD4+/CD8+ ratio was significantly correlated with treatment efficacy. The mean ratio and standard deviation of CD4 +/CD8 + ratio in all patients were 1.42 and 0.75 respectively. Patients who showed better responsiveness had a significantly higher proportion of CD4 +/CD8 + ratio than others (1.48 vs 1.25, respectively, p=0.059).

### Higher Peripheral CD4+ Was Significantly Associated With Improved Survival Outcomes For All Endpoints in mTNBC

**Figure 3:**
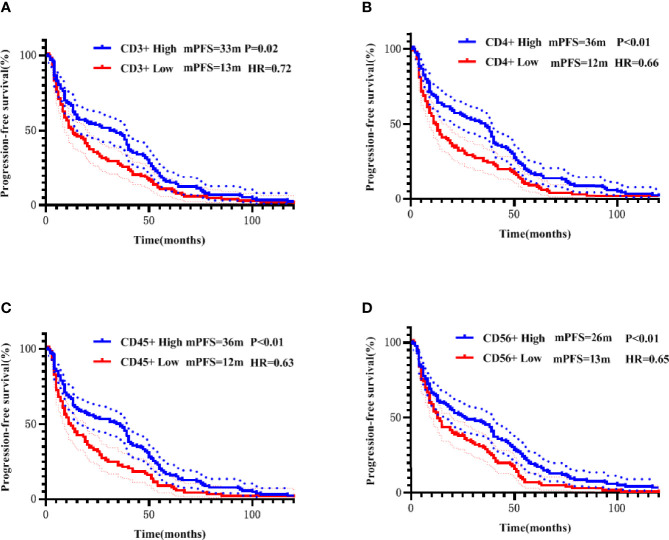
Correlation of CD3+ **(A)**, CD4+ **(B)**, CD45+ **(C)**, and CD56+ **(D)** with PFS addressed by the Kaplan-Meier method and log-rank test.

**Figure 4:**
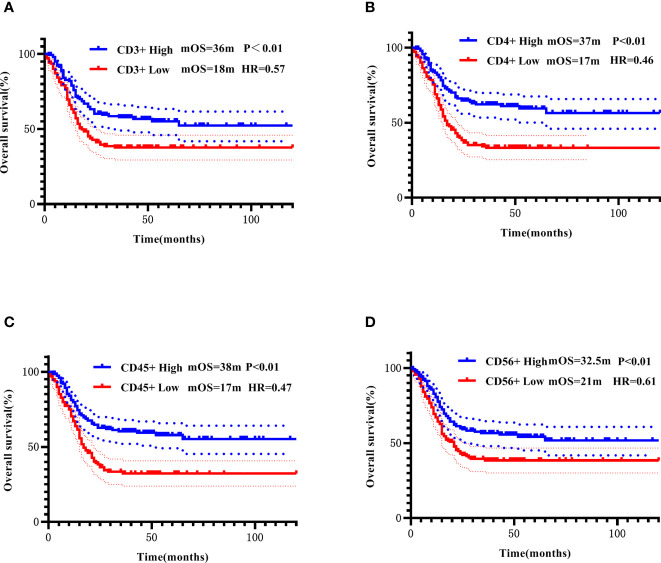
Correlation of CD3+ **(A)**, CD4+ **(B)**, CD45+ **(C)**, and CD56+ **(D)** with OS addressed by the Kaplan-Meier method and log-rank test.

For each PBL subgroup, taking the median as the cut-off value, each subgroup was divided into “high or low” subgroups. The median PFS of all patients was 11 months, and the median OS was 22 months. Kaplan-Meier analysis showed that higher subgroups of CD3 + (P = 0.02, **Figure 3A**), CD4 + (P < 0.01, **Figure 3B**), CD45 + (P < 0.01, **Figure 3C**) and CD56 + (P < 0.01, **Figure 3D**) were significantly associated with longer PFS. Similar correlation were observed significantly between higher subgroups of CD3 + (P < 0.01, **Figure 4A**), CD4 + (P < 0.01, **Figure 4B**), CD45 + (P < 0.01, **Figure 4C**) and CD56 + (P < 0.01, **Figure 4D**) and longer OS. In the multivariate Cox model, only CD4 + was found to be predictive with the median value of 540.00. Patients with a higher CD4 + showed both a significantly longer PFS (median PFS 36 months vs. 12 months; HR = 2.06, 95% CI: 1.145-3.7, P = 0.02) and OS (median OS 37 months vs. 17 months; HR = 2.21, 95% CI: 1.238-3.958, P< 0.01) compared with patients with a lower CD4 + (**Table 3A**).

**Table 3:** Multivariate prognostic value of circulating lymphocytes in TNBC for PFS and OS **(A)**, and multivariate prognostic value of circulating lymphocytes in mTNBC for PFS and OS **(B)**. Multivariate prognostic value of blood routine index, NLR, and PLR in TNBC for PFS and OS **(C)**.

A						
All Cases	PFS			OS		
	Multi P	95% CI	HR	Multi P	95% CI	HR
CD3+	0.275	0.371-1.325	0.702	0.232	0.36-1.282	0.679
CD4+	0.016	1.145-3.7	2.058	0.007	1.238-3.958	2.214
CD56+	0.630	0.714-1.746	1.116	0.409	0.765-1.934	1.216
CD45+	0.209	0.803-2.713	1.476	0.266	0.766-2.629	1.419
**B**						
**All Cases**	**PFS**			**OS**		
	Multi P	95% CI	HR	Multi P	95% CI	HR
CD3+	0.340	0.999-1.002	0.702	–	–	–
CD4+	0.010	0.440-0.973	0.650	0.032	0.417-0.940	0.630
CD45+	0.527	1.000-1.001	1.476	0.537	1.000-1.001	1.000
**C**						
**All Cases**	**PFS**			**OS**		
	Multi P	95% CI	HR	Multi P	95% CI	HR
White blood cells	0.524	0.565-1.338	0.869	–	–	–
Neutrophils	0.306	0.722-2.828	1.429	0.763	0.598-2.016	1.098
Lymphocytes	0.107	0.835-6.308	2.295	0.184	0.739-4.829	1.889
NLR	0.189	0.218-1.352	0.543	0.531	0.322-1.794	0.760
PLR	0.813	0.585-1.982	1.077	0.389	0.464-1.349	0.791

HR, hazard ratio,95%CI: 95% confidence interval.

PFS, progression-free survival; OS, overall survival.

Multi P, P-value for multivariate analysis. All P values were two-tailed-sided, and statistical significance was set as P < 0.05.

**Figure 5:**
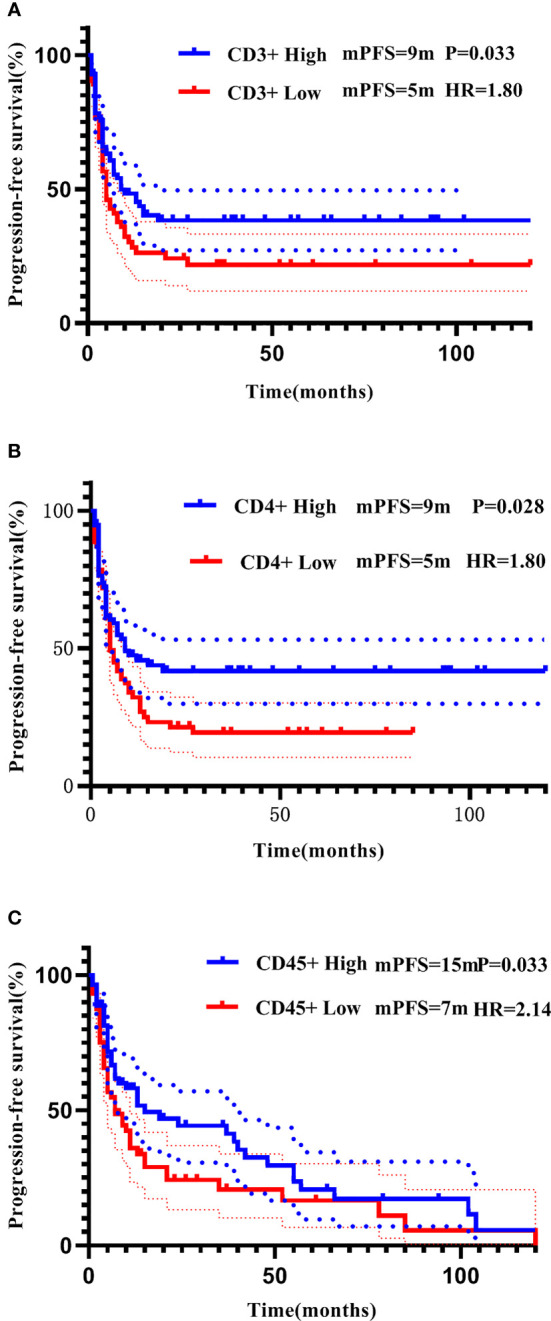
Correlation of CD3+ **(A)**, CD4+ **(B)**, and CD45+ **(C)** with PFS in patients with mTNBC addressed by the Kaplan-Meier method and log-rank test.

**Figure 6:**
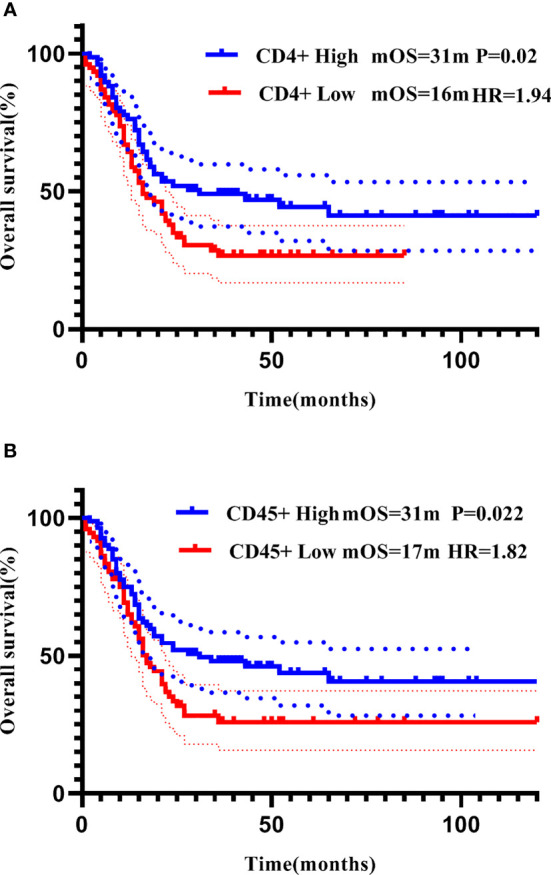
Correlation of CD4+ **(A)** and CD45+ **(B)** with OS in patients with mTNBC addressed by the Kaplan-Meier method and log-rank test.

All patients were further stratified into patients with early disease (including 92 patients receiving adjuvant therapy and 7 patients receiving neoadjuvant therapy) and mTNBC. All PBL subsets did not show prognostic significance in the early disease group. In the metastatic disease group, Kaplan-Meier analysis showed that higher CD3 +, CD4 +, and CD45 + were significantly associated with longer PFS (**Figures 5A–C**), higher CD4 +, and CD45 + were significantly associated with longer OS (**Figures 6A, B**). After incorporating the multivariate Cox model, still only CD4+ significantly predicted both PFS and OS (**Tables 3B**).

### Prognostic Analysis Of Blood Routine Indexes, NLR, and PLR

**Figure 7:**
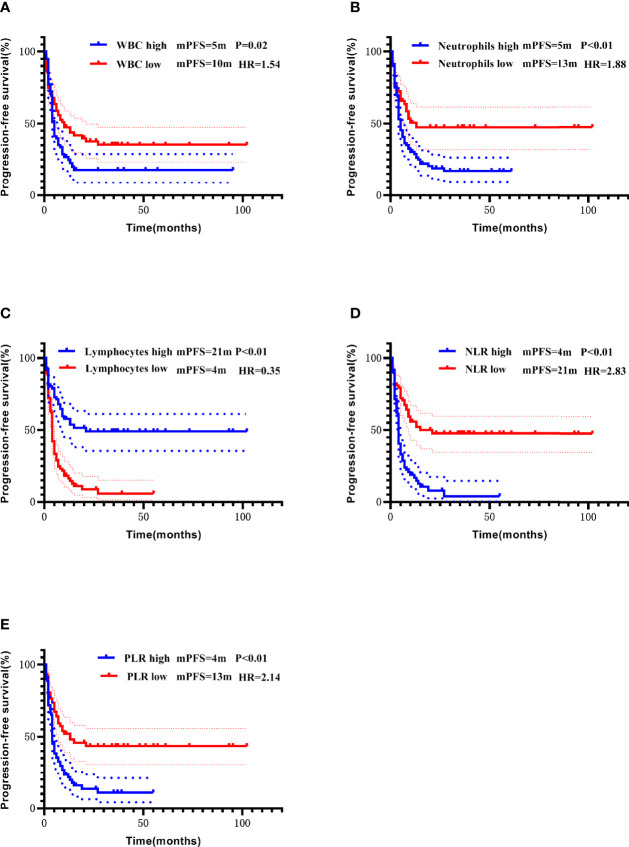
Correlation of white blood cells count **(A)**, neutrophils count **(B)**, lymphocytes count **(C**), NLR **(D)**, and PLR **(E)** with PFS addressed by the Kaplan-Meier method and log-rank test.

**Figure 8:**
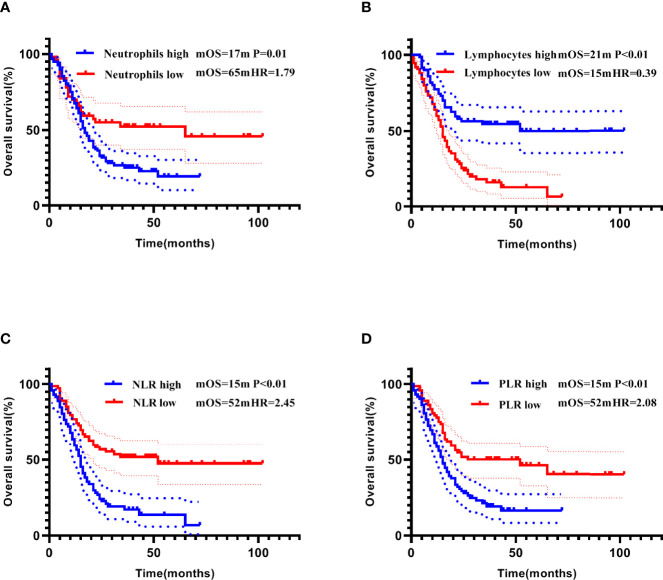
Correlation of neutrophils count **(A)**, lymphocytes count **(B)**, NLR **(C)**, and PLR **(D)** with OS addressed by the Kaplan-Meier method and log-rank test.

The median of each blood routine index, NLR, and PLR was used as the cut-off values to divide the patients into “high and low” subgroups. In the entire cohort, lower white blood cell count, neutrophil count, NLR, and PLR, and higher lymphocyte count were all significant predictors of longer PFS (Kaplan-Meier curves and log-rank test results in **Figures 7A–E**). Lower neutrophil count, NLR, and PLR, and higher lymphocyte count were significantly associated with longer OS (**Figures 8A–D**). However, in the multivariate Cox model, the above indicators failed to show statistical significance in predicting the prognosis (**Table 3C**).

### Relationship Between PBL Subsets and The Benefit Of Chemotherapy, Targeted Therapy, and ICIs Therapy in TNBC Patients

**Figure 9:**
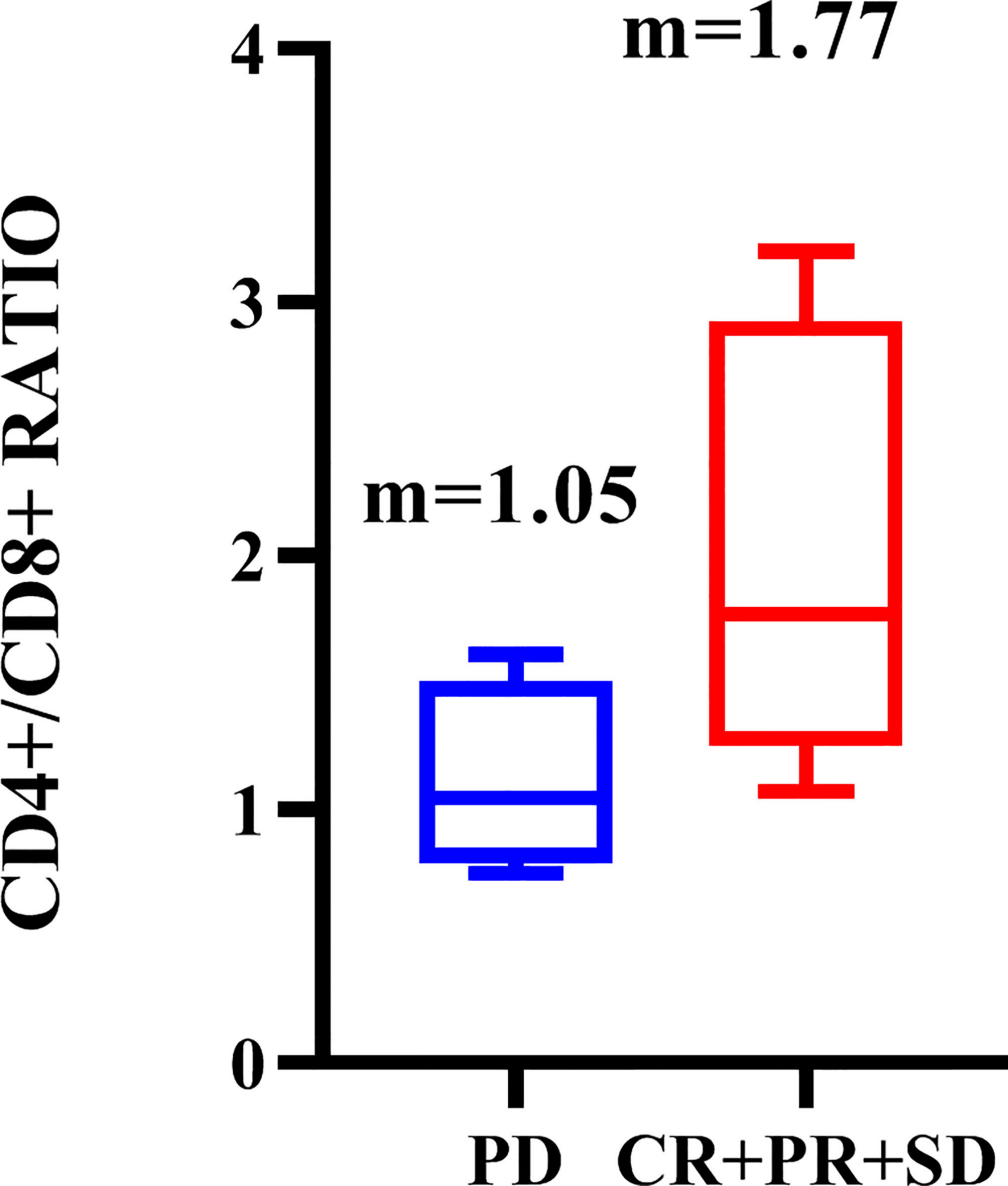
Distribution of peripheral blood CD4+/CD8+ in CR/PR/SD subgroup and PD subgroup in patients receiving chemotherapy combined with immunotherapy. M is the median of each lymphocyte subpopulation.

To investigate the predictive and prognostic impact of the immune-related markers across various treatments, we grouped all patients into chemotherapy only (n = 244), chemotherapy combined with targeted therapy (including vascular-targeted therapy n = 17 and PARP inhibitors therapy n = 3), and chemotherapy combined with ICIs therapy (including PD-1 inhibitors therapy n = 10, and PD-L1 inhibitors therapy n = 4). The correlation between PBL subgroup levels and the benefits of chemotherapy, targeted therapy, and ICIs therapy in patients with TNBC was observed in this study. Higher CD4 +/CD8 + ratio were significantly associated with a better response to chemotherapy combined with ICIs (P = 0.048, **Figure 9**). According to the stratified analysis of each treatment group, the CD4 + higher subgroup showed the tendency for a longer survival time (median PFS and OS reached versus 3 months). However, it only showed statistical significance in the chemotherapy subgroup (**Table 4**).

**Table 4:** Mann‐Whitney U test was conducted to detect differences in treatment response **(A)**, PFS **(B)**, and OS **(C)** among the chemotherapy only, chemotherapy combined with targeted therapy, and chemotherapy combined with immunotherapy subgroups.

A				
Treatments	Z		P	
Chemotherapy only	-1.278		0.201	
Chemotherapy combined with targeted therapy	-0.512		0.609	
Chemotherapy combined with immunotherapy	-1.98		0.048	
**B**				
**Treatments**	**mPFS (months)**		**Z**	**P**
	**CD4+ high**	**CD4+ low**		
Chemotherapy only	7	4	-2.129	0.033
Chemotherapy combined with targeted therapy	6	2	-1.79	0.073
Chemotherapy combined with immunotherapy	17	8	-1.63	0.103
**C**				
**Treatments**	**mOS (months)**		**Z**	**P**
	**CD4+ high**	**CD4+ low**		
Chemotherapy only	21	12.5	-3.007	0.002
Chemotherapy combined with targeted therapy	14.5	5	-1.641	0.101
Chemotherapy combined with immunotherapy	17	9	-1.133	0.257

mPFS, median of progression-free survival; mOS, median of overall survival.

All of the P values were two-tailed sided, and statistical significance was set as P < 0.05.

PFS and OS are displayed with the median of survival time.

### Clinicopathological Factors Associated With Peripheral CD4 + and CD4 +/CD8 + Ratio

Considering that the clinicopathological parameters may affect the distribution of PBL subsets, we analyzed the correlations of PBL subsets with clinicopathological parameters, such as Ki-67, Karnofsky Performance Status (KPS), menstrual status, age, BRCA status, advanced treatment line, type of treatment, neoadjuvant/adjuvant therapy use, metastasis location and status, tumor size, lymph node metastasis status, and clinical stage. As shown in **Table 5**, the CD4 +/CD8 + ratio and CD4+ were significantly lower in the age ≤50 group than in the age>50 group. The CD4 +/CD8 + ratio level was significantly lower in the group receiving neoadjuvant therapy than in the group not receiving neoadjuvant therapy (P = 0.027). The higher clinical stage (P = 0.001) and more advanced treatment lines (P < 0.001) were significantly related to the lower level of CD4+ in patients with TNBC.

**Table 5:** Spearman’s Rho and Mann-Whitney test for comparison of clinicopathological parameters according to peripheral CD4+ and CD4+/CD8+ in TNBC.

Clinicopathological characteristic	CD4+	P value	CD4+/CD8+ Ratio	P value
**Age(y)**		0.014		0.01
≤50	560.45 ± 393.92		1.49 ± 0.75	
>50	645.72 ± 324.37		1.68 ± 0.75	
**Ki67**		0.662		0.48
Positive(≥14%)	575.84 ± 363.19		1.57 ± 0.78	
Negative(<14%)	680.76 ± 406.85		1.65 ± 0.68	
**Pathologic T stage(Tumor size)**		0.17		0.539
1	679.89 ± 425.31		1.67 ± 0.81	
2-4	556.96 ± 316.83		1.52 ± 0.72	
**Pathologic N stage**		0.392		0.294
0	650.74 ± 434.27		1.55 ± 0.71	
1-2	571.00 ± 291.57		1.63 ± 0.79	
3	530.34 ± 257.78		1.43 ± 0.75	
**Clinical stages**		0.001		0.237
0-1	725.91 ± 507.29		1.62 ± 0.75	
2	677.97 ± 366.20		1.67 ± 0.93	
3	698.36 ± 355.13		1.83 ± 0.83	
4	522.24 ± 264.38		1.50 ± 0.71	
**Bone invasion**		0.735		0.735
Yes	527.81 ± 126.51		1.66 ± 0.68	
No	603.34 ± 368.81		1.57 ± 0.76	
**Visceral invasion**		0.097		0.702
Yes	546.62 ± 297.20		1.55 ± 0.76	
No	627.31 ± 389.96		1.59 ± 0.75	
**Multiple invasion**		0.316		0.802
Yes	551.70 ± 304.16		1.53 ± 0.73	
No	627.68 ± 390.48		1.60 ± 0.76	
**Neoadjuvant therapy**		0.057		0.027
Yes	486.95 ± 268.77		1.34 ± 0.62	
No	624.68 ± 376.63		1.63 ± 0.77	
**Type of neoadjuvant treatment**		0.445		0.18
Chemotherapy only	486.68 ± 271.67		1.33 ± 0.62	
Chemotherapy combined with targeted therapy	–		–	
Chemotherapy combined with immunotherapy	–		–	
**Postoperative adjuvant therapy**		0.246		0.619
Yes	602.72 ± 359.40		1.59 ± 0.77	
No	590.93 ± 390.45		1.53 ± 0.69	
**Type of adjuvant treatment**		0.3		0.325
Chemotherapy only	607.20 ± 359.70		1.59 ± 0.77	
Chemotherapy combined with targeted therapy	179.36		1.58	
Chemotherapy combined with immunotherapy	–		–	
**Advanced treatment lines**		<0.001		0.127
1	564.65 ± 301.96		1.54 ± 0.73	
2	571.29 ± 295.23		1.62 ± 0.74	
≥3	425.48 ± 234.31		1.46 ± 0.79	
**Type of advanced treatment**		0.601		0.756
Chemotherapy only	546.07 ± 267.16		1.53 ± 0.74	
Chemotherapy combined with targeted therapy	642.69 ± 420.000		1.50 ± 0.76	
Chemotherapy combined with Immunotherapy	577.42 ± 436.67		1.71 ± 0.79	
**BRCA status**		0.48		0.643
Mutation	387.62 ± 362.29		0.87 ± 0.52	
Non-mutation	238.44 ± 259.65		1.04 ± 0.18	
Unknown	608.06 ± 362.68		1.59 ± 0.75	
**Family tumor history**		0.632		0.632
Yes	678.05 ± 570.60		1.56 ± 0.78	
No	585.20 ± 304.46		1.58 ± 0.75	
**Menstrual status**		0.551		0.055
Premenopause	634.84 ± 477.04		1.54 ± 0.80	
Postmenopause	613.31 ± 323.27		1.59 ± 0.71	
**KPS**		0.365		0.146
≥80	609.31 ± 377.60		1.59 ± 0.75	
50-70	235.76 ± 201.17		1.29 ± 0.82	

KPS, Karnofsky Performance Status.

Visceral metastasis includes the brain, liver, and lung metastasis. Multiple lesions occurred in the same organ only count once. The median value of lymphocytes was used as the cut-off value.

All P values were two-tailed-sided, and statistical significance was set as P < 0.05.

## Discussion

To the best of our knowledge, this study was the first one aimed at patients with TNBC, demonstrating that peripheral blood lymphocyte subsets could significantly represent both predictive and prognostic value. Indeed, pre-treatment peripheral CD4+ was found to be a strong positive predictor of all clinical outcomes in our cohort of mTNBC patients, especially those who accepted chemotherapy. Another important finding was that higher CD4+/CD8+ could predict better treatment responsiveness of patients with TNBC.

Within lymphocyte subsets, higher baseline CD3 +, CD4 +, CD56 +, and CD45 + in peripheral blood correlate slightly with a better PFS and OS in univariate analysis, but only CD4+ maintained prognostics value in a multivariate Cox model. Stratifying patients across early and late stages of TNBC for prognostic analysis, CD4+ maintained a stable predictive significance for both PFS and OS in patients with mTNBC. Contrary to expectations, we did not find any PBL subset that significantly predicted PFS or OS in patients with early disease. These results seem to be consistent with another research that analyzed patients with MBC, included 157 patients with TNBC, and found the decrease of pre-treatment CD4 + in peripheral blood was significantly related to the poor prognosis (16). In addition, an abundance of studies explored the prognostic significance of TILs in patients with TNBC. A meta-analysis systematically reviewed 37 studies and concluded that high CD4 + TILs levels were associated with better DFS and OS (17). Another study constructed an immune phenotype classifier for predicting clinical prognosis and immune activity in TNBC, enrolling a total of 770 patients, and found that stormal CD4+ T cells predicted better prognosis (18). Furthermore, Kwan Ho Lee et al. and Lei Wang et al. provided important insights into the connection between the immunocompetence of PBL and intratumoral T cells. These findings also supported our results to some extent (19, 20). Other studies by Jian Yang et al. and Xiao-Ran Liu et al. suggested that plasma CD4+ and peripheral cytotoxic T lymphocyte (pCTL) were negative independent predictors of PFS in HER-2 positive patients. However, in their study, CD4+ and pCTL did not show the predictive significance of OS and had a poor indication for TNBC (21, 22). There was no clear biological explanation for the different prognoses of PBL subsets in different breast cancer subtypes. Interestingly, the opposite effects of TILs on survival for TNBC and HER2-positive breast cancer were also found in a pooled analysis of 3771 BC patients treated with neoadjuvant therapy (9). A possible explanation for this might be that they found the composition and magnitude of the tumor immune infiltrate varied with different breast cancer subtypes. Further work is required to establish the various impacts of these PBL subsets on the prognosis in different breast cancer subtypes.

Several reports have shown that various blood cell counts and the ratios between them, such as NLR and PLR, could predict survival in breast cancer. Most of them reported that peripheral lymphocyte count was a favorable prognostic factor (23), while NLR and PLR were unfavorable prognostic factors in patients with TNBC (24–27). Our results were in accord with earlier studies indicating that higher lymphocytes count was associated with a good prognosis, higher neutrophils count, NLR, and PLR were associated with poor prognosis in patients with TNBC, although the difference was not statistically significant in a multivariate analysis.

We further performed a stratified analysis of various treatment methods, came to a similar conclusion with Robert et al. that peripheral CD4 + had a substantial prognostic effect on patients with TNBC who received chemotherapy (28). Interestingly, many previous studies have proposed that the immune status may be affected by the specific chemotherapy regimen and the mode of administration. Since our sample includes multi-line and cross-line treatments, we didn’t count the particular chemotherapy regimen data. It might be possible to analyze whether different chemotherapy regimens can identify different PBL subsets as prognostic biomarkers in future investigations. In the subgroups of chemotherapy combined with targeted therapy and ICIs, We also observed that the peripheral CD4 + higher subgroup showed the trend of longer PFS and OS than the CD4 + lower subgroup. Contrary to expectations, the results did not reach statistical differences. This may be due to the limitation of the existing medical condition that few patients were receiving targeted therapy and ICIs therapy in our sample. Recently, a study reported consistently with our results that higher baseline CD4+ T cells proportion in the blood is potential biomarkers for combinational anti-pathogenesis and immunotherapy in advanced TNBC patients (29). However, in the past exploration, CD8+ was considered an essential predictor of TNBC, and CD8+ TILs evaluation as a stratified feature in an immunotherapy phase III trial (Impassion130) could suggest a better clinical outcome (30). In contrast, this significance was not observed in our results. Therefore, there is a need to further expand the sample to analyze specific populations treated with targeted therapy and ICIs therapy.

By analyzing the contribution of individual blood routine indexes and PBL subsets to treatment responsiveness, a significant correlation was found between clinical benefit and higher CD4+/CD8+ in patients with TNBC. The same change of CD4+/CD8+ in TILs was also found to be associated with therapeutic response or tumor progression in breast cancer patients who received neoadjuvant chemotherapy (31, 32). Surprisingly, CD4 +/CD8 + had a significant predictive value for subgroups treated with chemotherapy combined with immune checkpoint inhibitors in our cohort. A systematic review included 27 findings in 1746 patients with MBC and found high TIL and CD8+ T-cell infiltrating levels could predict better response to ICI treatment (33). However, in our study, peripheral CD8+ T cells did not show the predictive significance for the response to ICIs treatment. Although a large amount of research has been carried out, there was no clinically precise biomarker able to select responsive or resistant patients treated with ICIs. The response to immunotherapy was considered to depend on the dynamic interaction between tumor and immune cells in the tumor microenvironment. This observation advised cautiously that peripheral CD4 +/CD8 + might have a suggestive effect on predicting the response to immunotherapy in patients with TNBC. However, with small sample size, the results must be further verified in the population of TNBC patients receiving ICIs therapy in the future.

Furthermore, we analyzed the correlation between the distributions of PBL subsets and clinicopathological parameters in patients with TNBC. The data showed that lower CD4+ and CD4+/CD8+ were related to an older age. Patients who had received neoadjuvant therapy presented a higher CD4 +/CD8 + ratio. For CD4+, it was correlated with clinical stage and advanced treatment lines. The definite relationship between lymphocyte subsets and clinicopathological factors should be revealed in future investigations.

Nevertheless, limited by the detection method, we were unable to further evaluate the frequency of CD4 + T cell subsets. Previous reports have demonstrated that the phenotype of lymphocyte infiltrates also determines clinical outcomes (34). CD4 + T-helper 1 (Th1) cells perform antigen presentation by promoting cytokine secretion and antigen-presenting cell activation (35). Th1 cells were considered as a favorable prognostic factor (36). On the other hand, CD4 + T-helper 2 (Th2) cells, including Foxp3+ Treg cells, inhibit CTL function, support B lymphocyte proliferation, and may promote anti-inflammatory immune response, thereby promoting tumor growth (37, 38). The presence of Tregs has been associated with both good and bad prognoses. Among other CD4 + T cell subsets, Th17 cells are the producers of the pro-inflammatory cytokine family IL-17. They seem to have different roles according to the surrounding cytokine environment, which may be partly related to organ location and tumor type (39). Follicular helper T cells (TFH) are the latest reported CD4 + subsets positively correlated with the prognosis of patients in adjuvant chemotherapy and neoadjuvant chemotherapy (40). There is abundant room for further progress in determining the prognostic role of specific CD4+ subsets in TNBC.

## Conclusion

In conclusion, we found that the baseline CD4+ level in the peripheral blood of TNBC patients was significantly associated with PFS and OS, particularly those treated with chemotherapy. The peripheral CD4+/CD8+ ratio could predict therapeutic response, especially to immunotherapy. And the alteration of PBL subsets was closely related to clinicopathological factors in TNBC patients. As an easy-assessable and less-invasive approach, the detection of peripheral blood lymphocyte subsets may further stratify TNBC patients in clinical treatment involving chemotherapy and future combinations with immune therapies. It will be interesting to further explore peripheral lymphocyte subsets in connection with TILs as a potential biomarker for treatment efficacy and survival in future clinical Trials.

## Data Availability Statement

The original contributions presented in the study are included in the article/supplementary material. Further inquiries can be directed to the corresponding author.

## Ethics Statement

This article is approved by the ethics committee of our institution with the ethics number 20201133K.

## Author Contributions

All authors contributed to the article and approved the submitted version. ML and JX were responsible for conceiving and designing the study. ML performed the study, analyzed the data, and wrote the manuscript. JX, CJ and TS critically revised the manuscript and ensured correct data analysis. JZ assisted in data collection and analysis.

## Conflict of Interest

The authors declare that the research was conducted in the absence of any commercial or financial relationships that could be construed as a potential conflict of interest.

## Publisher’s Note

All claims expressed in this article are solely those of the authors and do not necessarily represent those of their affiliated organizations, or those of the publisher, the editors and the reviewers. Any product that may be evaluated in this article, or claim that may be made by its manufacturer, is not guaranteed or endorsed by the publisher.
